# Adsorption of Toxic Metals Using Hydrous Ferric Oxide Nanoparticles Embedded in Hybrid Ion-Exchange Resins

**DOI:** 10.3390/ma17051168

**Published:** 2024-03-01

**Authors:** Zizikazi Sodzidzi, Zebron Phiri, Jemal Fito Nure, Titus A. M. Msagati, Lueta-Ann de Kock

**Affiliations:** Institute for Nanotechnology and Water Sustainability (iNanoWS), College of Science Engineering and Technology, University of South Africa, Florida Science Campus, Johannesburg 1709, South Africa; zizikazis@gmail.com (Z.S.); enurejf@unisa.ac.za (J.F.N.); msagatam@unisa.ac.za (T.A.M.M.); dkockla@unisa.ac.za (L.-A.d.K.)

**Keywords:** acid mine drainage, adsorption, hybrid ion-exchange resins, hydrous ferric oxide nanoparticles, toxic heavy metals

## Abstract

Acid mine drainage (AMD) is a major environmental problem caused by the release of acidic, toxic, and sulfate-rich water from mining sites. This study aimed to develop novel adsorbents for the removal of chromium (Cr(VI)), cadmium (Cd(II)), and lead (Pb(II)) from simulated and actual AMD using hybrid ion-exchange resins embedded with hydrous ferric oxide (HFO). Two types of resins were synthesized: anionic exchange resin (HAIX-HFO) for Cr(VI) removal and cationic exchange resin (HCIX-HFO) for Cd(II) and Pb(II) removal. The resins were characterized using scanning electron microscopy and Raman spectroscopy, which confirmed the presence of HFO particles. Batch adsorption experiments were conducted under acidic and sulfate-enhanced conditions to evaluate the adsorption capacity and kinetics of the resins. It was found that both resins exhibited high adsorption efficiencies and fast adsorption rates for their respective metal ions. To explore the potential adsorption on actual AMD, HCIX-HFO demonstrated significant removal of some metal ions. The saturated HCIX-HFO resin was regenerated using NaCl, and a high amount of the adsorbed Cd(II) and Pb(II) was recovered. This study demonstrates that HFO-embedded hybrid ion-exchange resins are promising adsorbents for treating AMD contaminated with heavy metals.

## 1. Introduction

Mining is an essential driver of economic growth and prosperity for many countries. However, mining also poses serious environmental challenges, especially in the form of heavy metal contamination from acid mine drainage (AMD). AMD is the acidic and metal-rich water that results from the oxidation of sulfide-containing minerals exposed during mining activities [1,2]. AMD can pollute nearby water resources, endangering the ecosystem and human health [3,4]. Heavy metals are persistent in the environment and can accumulate in soft tissues, disrupting the normal functioning of vital organs and systems [5,6]. While mineral exploration drives economic advancement, addressing the potential environmental implications is essential to ensure sustainable development.

Treatment of AMD is a costly exercise due to the complex and heterogeneous nature of its composition, typical of its origin [7]. Researchers have explored various techniques to treat AMD, such as chemical precipitation [8], membrane filtration [9], electrochemical treatment [10], and biological remediation [11]. However, these methods have drawbacks, such as low efficiency, high requirements of chemical inputs, and secondary pollution [12]. Therefore, there is a need for novel strategies that can improve AMD treatment and remove heavy metals more effectively. Adsorption and ion exchange are promising heavy metal remediation techniques [12,13,14]. Adsorption is a simple, versatile, and cost-effective technique that involves the attachment of contaminants onto the surface of an adsorbent. On the other hand, ion exchange is a technique that uses engineered polymers with fixed charges and mobile counter-ions to exchange ions with the solution based on their affinities [15]. Both techniques provide reusability without significant performance loss, making them attractive options for heavy metals removal in aquatic media [16,17,18].

Various organic and inorganic adsorbents such as activated carbon, zerovalent iron, metal-organic frameworks, clay minerals, zeolites, layered double hydroxides, and modified biochar have been proposed [19,20,21]. These adsorbents can remove heavy metals from wastewater using different mechanisms, such as adsorption, reduction, precipitation, and complexation. However, these adsorbents also have drawbacks regarding their selectivity, capacity, and stability, limiting their applicability and efficiency for heavy metal remediation. For instance, metal-organic frameworks are expensive and sensitive to pH changes and water stability [22]. Clay minerals have a low selectivity for metal ions and can be affected by competing anions [23]. Zeolites have a narrow pH range for optimal performance and can be saturated quickly [24].

Improving the properties and performance of these adsorbents or developing new materials that can overcome these limitations for AMD remediation when necessary; hence, the growing interest in novel adsorbents that can enhance the adsorption processes. Researchers have explored using metal oxide nanoparticles as efficient adsorbents for heavy metal removal [25]. Metal oxide nanoparticles have a high surface area and reactivity, which enhance their adsorption capacity and selectivity [26,27,28]. However, the sole usage of nanoparticles presents practical challenges, such as aggregation and instability [29,30]. On the other hand, ion-exchange resins utilize the Donnan membrane effect, where the fixed charge in the ion-exchange resin attracts the corresponding opposite-charged ions to the pores of the resin [31,32]. However, ion-exchange resins possess limited selectivity and capacity for particular ions, limiting their adsorption efficiency [33]. The reduced adsorption capacity usually occurs due to competing ions, resulting from the simultaneous adsorption of many different ions.

To overcome these challenges, researchers have incorporated nanoparticles into polymeric matrices, such as ion-exchange resins, to create hybrid materials that combine the advantages of both components [34,35]. The performance of ion exchangers modified with hydrous ferric oxide (HFO) nanoparticles for phosphate removal from wastewater has been studied by various researchers [36,37,38]. Gifford et al. [39] delved into the performance of HFO or titanium dioxide nanoparticles precipitated within anion-exchange resins, targeting the removal of chromium and arsenic. Nguyen et al. [40] prepared a composite of cation-exchange resin-supported iron and magnesium oxides/hydroxides as a potential adsorbent for nitrate ions from water. Maltseva et al. [41] demonstrated that the sorption of arsenate ions was enhanced via ion exchangers modified with hydrated oxides of zirconium and iron.

One of the main challenges of treating AMD is its high concentration of sulfate ions, which can interfere with the removal of Cr(VI). With its divalent negative charge, sulfate challenges Cr(VI) in competing for anion-exchange resin sites. Previous studies, such as Acelas et al. [42], have highlighted the significant negative effect of SO_4_^2−^ on phosphate adsorption using a hybrid anion exchanger with embedded iron oxide. Other researchers, such as Hua et al. [43], Kolodynska et al. [44], and Kowalczyk et al. [45], have investigated the delicate balance of sulfates and other anions in the adsorption of Cr(VI) using anion exchangers with embedded HFO. However, these studies used low SO_4_^2−^ concentrations (40–50 mg·L^−1^). In comparison, the actual sulfate concentration in AMD from the Witwatersrand Mining Basin in South Africa ranges from 1500 mg·L^−1^ to 3000 mg·L^−1^ [46]. This significant difference sets the basis for this study, which aimed to simulate the high sulfate levels in AMD.

This study investigated the potential of ion-exchange resins and hydrous ferric oxide (HFO) as an adsorbent that combines two adsorption sites to remove heavy metals. HFO is a natural mineral that can form precipitates or coatings on various substrates and has a high affinity for metal ions [47,48]. Two types of hybrid resins were synthesized: anion-exchange resin (HAIX-HFO) for chromium (Cr(VI)) removal and cation-exchange resin (HCIX-HFO) for cadmium (Cd(II)) and lead (Pb(II)) removal in acidic and high sulfate conditions. The physicochemical properties of these hybrid resins were characterized, and their adsorption performance was evaluated under different experimental conditions. Furthermore, the feasibility study of regenerating and reusing these hybrid resins after saturation was investigated. The synthesized adsorbents have the potential for the remediation of heavy metals in aqueous environments.

## 2. Materials and Methods

### 2.1. Materials and Standards

Unless otherwise stated, all chemical reagents used in this study were of analytical grade and obtained from Sigma Aldrich. The hybrid ion-exchange resins embedded with hydrous ferric oxides (HFOs) were synthesized using FeCl_3_·6H_2_O, 32% HCL, 65% HNO_3_, NaOH, and NaCl. Before the analysis, inductively coupled plasma optical emission spectroscopy (ICP-OES) was calibrated using a multi-elemental standard. The host resins, Amberlite IRA400 Cl form (HAIX) and IMAC HP 1110 (HCIX), were obtained from Sigma Aldrich (Merck, Johannesburg, South Africa) and Lenntech (Delfgauw, The Netherlands), respectively. As shown in Table 1, Amberlite IRA400 Cl form is a gel-type, strong basic anion-exchange resin, while IMAC HP1110 is a strong acid cation resin. For the adsorption studies, K_2_Cr_2_O_7_, CdSO_4_·H_2_O, and PbSO_4_ were utilized, and the respective sulfate solutions were prepared using Na_2_SO_4_ (Rochelle chemicals).

### 2.2. Analytical Techniques for Characterization

To investigate the surface morphology and elemental composition of the dispersed nanoparticles in the hybrid ion-exchange resins, a field emission scanning electron microscope (FESEM) (model JEOL JSM-7800F), coupled with energy-dispersive X-ray spectroscopy (EDS) (Thermo Scientific Ultradry) detector was used. The FESEM was operated at an accelerating voltage of 5 kV, a magnification of 900–9500×, a resolution of 1–10 µm, and a scan time of 10 s. The EDS spectra were collected using an acquisition time of 60 s and a dead time of less than 25%.

Raman spectroscopy (Witec, Alpha 300, TS 150 Raman spectrometer), with a laser power source of 532 mW and 784.898 mW as an excitation source, was used to identify the form of the iron oxide in the hybrid ion-exchange resins. The Raman spectra were recorded in the range of 100–2000 cm^−1^, with a resolution of 4 cm^−1^, and an integration time of 10 s.

The Agilent Technologies 700 series ICP-OES was used to analyze the qualitative and quantitative levels of metals in aqueous samples. The following conditions were used to operate the ICP-OES: an RF power of 1500 W, a plasma gas maintained at a flow rate of 15 L·min^−1^, an auxiliary gas flow rate kept at 1.5 L·min^−1^, a nebulizer gas flow rate of 0.8 L·min^−1^, a sample uptake rate of 1 mL·min^−1^, and an integration time of 10 s per replicate.

### 2.3. Synthesis of the Hybrid Ion-Exchange Resins Embedded with Hydrous Ferric Oxide Nanoparticles

Anionic and cationic hybrid ion-exchange resins embedded with HFO nanoparticles were synthesized using a method prescribed by Pan and colleagues [49]. The synthesis steps were conducted using a thermostatic shaker that maintained a shaking speed of 200 rpm and 25–26 °C temperature range. The synthesis of respective hybrid exchange resins (anionic or cationic) embedded with HFO nanoparticles entailed washing 100 g of Amberlite IRA 400 Cl form or IMAC HP 1110 with 500 mL of deionized water through shaking for two hours to remove fine particles and any residual organic material.

To lodge HFO into HAIX resins, the ferric chloride anionic complex was prepared by dissolving 135.42 g of FeCl_3_·6H_2_O in 500 mL of 1 M HCl to produce Fe^3+^ on the quaternary nitrogen sites of the Amberlite IRA 400 Cl form. The hydrolysis reaction formed [Fe(H_2_O)_6_]^3+^ was subsequently converted to HFO nanoparticles embedded in the resin matrix. To increase the yield, the prepared solution and the rinsed Amberlite IRA 400 Cl form were agitated in a thermostatic shaker for 24 h.

To embed HFO nanoparticles into HCIX resin, 135.42 g of FeCl_3_·6H_2_O was dissolved in 500 mL of deionized water: the Fe^3+^ exchanges with the Na^+^ counter ion of the cation-exchange resin. The FeCl_3_ solution was added to the rinsed IMAC HP 1110 resin and shook it in a thermostatic shaker for 24 h. Respective FeCl_4_^−^ and FeCl_3_ solutions were decanted, followed by the addition of 300 mL 1 M NaOH aliquots and shaking while measuring the pH every 10 min using a pH meter. The solution was decanted at pH 12.

Subsequently, both resins were placed in an oven at 40 °C for 24 h. This step was followed by cooling the resins to room temperature and then rinsing with 300 mL aliquots of 1 M NaCl until the pH meter showed pH 7. After decanting the supernatant, the resins were air-dried to form HAIX-HFO and HCIX-HFO.

### 2.4. Adsorption Studies

This study investigated the batch adsorption of Cr(VI), Cd(II), and Pb(II) on two hybrid ion-exchange resins: HCIX-HFO and HAIX-HFO. The effects of various parameters, such as pH, sulfate concentration, contact time, and adsorbent dosage, on the removal efficiency of the three metals were examined. The adsorption data were fitted to kinetic and isotherm models to elucidate the adsorption mechanisms. Additionally, this study explored the impact of sulfate on the adsorption of metal ions and the competition between Cd(II) and Pb(II) in binary solution for the available adsorption sites. The performance of HCIX-HFO in treating actual AMD samples was also evaluated. Moreover, batch desorption studies were conducted to assess the feasibility of regenerating the heavy metal-loaded HCIX-HFO.

#### 2.4.1. Effect of pH, Sulfate Concentration, Contact Time, and Resin Dosage

The impacts of varying pH levels on adsorption were examined. Solutions containing Cr(VI), Cd(II)), and lead (Pb(II)) at a concentration of 1 mg·L^−1^ were adjusted to pH values ranging from 2 to 5 using either HCl or NaOH. Subsequently, 0.005 g of HAIX-HFO was added to the Cr(VI) solutions, while 0.005 g of HCIX-HFO was added to the Cd(II) and Pb(II) solutions.

The effects of sulfate concentrations, mimicking typical levels found in AMDs, were explored. Solutions containing 1 mg·L^−1^ of Cr(VI) were prepared with varying sulfate concentrations ranging from 0 to 3000 mg·L^−1^ [46,50,51]. The pH of the Cr(VI) solution was adjusted to 4 before being added to HAIX-HFO resin. This step and the effects of pH involved 24 h of 200 rpm agitation.

The influence of contact time on adsorption was further investigated. A Cr(VI) solution at a concentration of 1 mg·L^−1^ and a pH of 4 was prepared and agitated with the HAIX-HFO resin for various time intervals ranging from 5 to 360 min.

Lastly, the effect of varying the dosage of the HAIX-HFO resin on the adsorption of Cr(VI) was examined. Solutions containing Cr(VI) at a concentration of 1 mg·L^−1^ and a pH of 4 were prepared and agitated with different amounts of the HAIX-HFO resin ranging from 0.001 g to 0.01 g for 360 min. All steps involved maintaining the temperature at 25–26 °C, filtering the mixtures, and analyzing the remaining metal concentrations using ICP-OES.

A similar approach for studying the effects of sulfate concentration, contact time, and hybrid resin dosage on Cd(II) and Pb(II) adsorption was employed. However, these studies used the HCIX-HFO resin instead of the HAIX-HFO resin. Also, the competitive adsorption values of Cd(II) and Pb(II) were determined. A binary stock solution that contained 1 mg·L^−1^ Cd(II), 1 mg·L^−1^ Pb(II), and 3000 mg·L^−1^ sulfate was prepared and adjusted to pH 4. Then, 25 mL aliquots of the binary solution were added to 0.005 g portions of the HCIX-HFO resin and placed in a shaker for 24 h at 25–26 °C. The mixtures were filtered, and the remaining Cd(II) and Pb(II) concentrations in the supernatants were determined via ICP-OES.

#### 2.4.2. AMD Remediation

An actual AMD sample from the Western Witwatersrand Mining Basin (Johannesburg, South Africa) was used in this study. The pH of the sample was 2.62, and its metal composition was determined via ICP-OES (Table 2). Evaluation of the adsorption of heavy metals with the HCIX-HFO resin was performed by adding 25 mL of the AMD sample to 0.005 g of the HCIX-HFO resin and shaking it at 200 rpm for 360 min at 25–26 °C. Filtered solutions were measured via ICP-OES to determine the concentration of metal species.

#### 2.4.3. Resin Regeneration

Regeneration of Pb(II) or Cd(II) metal-laden HCIX-HFO was evaluated using NaCl and NaOH. The saturated mixture of the HCIX-HFO resin with either 5% NaCl or 1 M NaOH solution was agitated for 24 h at 25–26 °C. The mixtures were filtered, and the supernatants were analyzed for Pb(II) and Cd(II) concentrations using ICP-OES.

## 3. Results and Discussion

### 3.1. Characterization

**Figure 1:**
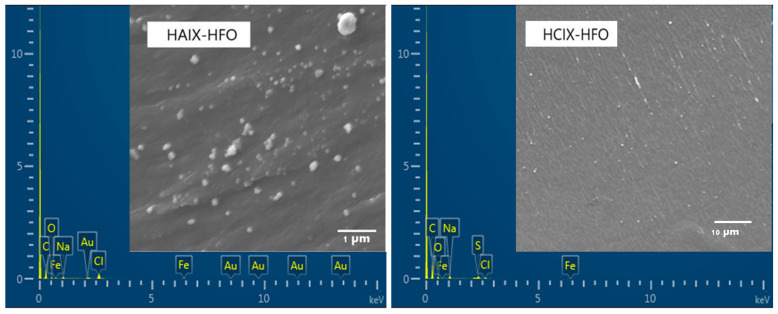
SEM images and EDS spectra of the hybrid anionic and cationic exchange resins embedded with HFO.

The hybrid resins HAIX-HFO and HCIX-HFO were characterized using SEM-EDS and Raman spectroscopy to determine the distribution, composition, and type of iron oxide nanoparticles embedded in the resin beads. Their cross-sectional surfaces were examined using SEM-EDS at different magnifications (Figure 1) and observed white spots, which are HFO nanoparticles, almost evenly distributed throughout the interior of HAIX-HFO. This observation suggests that there was a minimal agglomeration of HFO particles. A uniform incorporation of HFO nanoparticles in HCIX-HFO was also observed, although some agglomeration was evident.

EDS spectra confirmed the presence of both HFO nanoparticles and Fe in the resins. For HAIX-HFO, Fe bands at 0.7 and 6.5 keV indicated HFO incorporation, while Cl bands demonstrated that the quaternary ammonium ion-exchange sites were in chloride form. The weight % of Fe in HAIX-HFO was 4.4%, lower than the 14.0% reported by De Kock [52]. For HCIX-HFO, Fe bands at 0.6 and 6.5 keV also confirmed HFO incorporation, while Na bands revealed that the sulfonate ion-exchange sites were in sodium form. The weight % of Fe in HCIX-HFO was 14.9%, comparable to a previous study [52].

**Figure 2:**
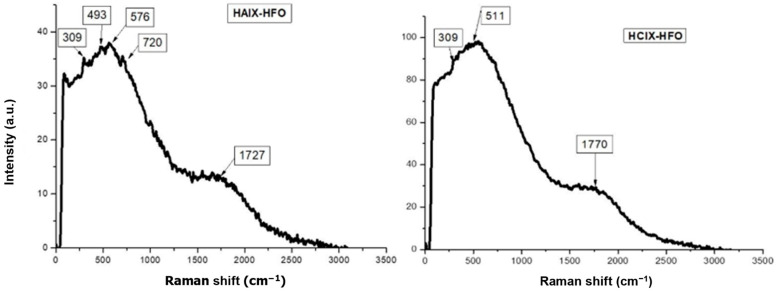
Raman spectra for HAIX-HFO and HCIX-HFO resin beads.

The iron oxide types in HAIX-HFO and HCIX-HFO were identified using Raman spectroscopy (Figure 2), and the iron oxide phase in both hybrid resins was found to be ferrihydrite. The band at 720 cm^−1^ for HAIX-HFO and 511 cm^−1^ for HCIX-HFO matched the characteristic bands of ferrihydrite reported in the literature [53,54,55]. However, it is noteworthy that the fluorescence of the organic backbone of the resin matrix affects the Raman spectra [56,57], which obscures some bands and makes identifying the iron oxide phase challenging.

### 3.2. Batch Adsorption Studies

#### 3.2.1. Effects of pH

**Figure 3:**
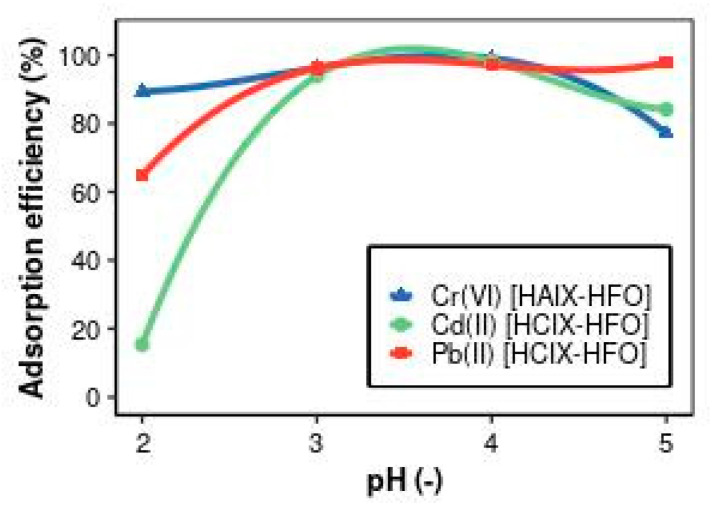
Effects of pH on removal adsorption efficiency of Cr(VI), Cd(II), and Pb(II).

HAIX-HFO immobilizes HCrO_4_^−^ or Cr_2_O_7_^2−^ forms of Cr(VI) ions at low pH values. Figure 3 shows that the removal efficiency of Cr(VI) with HAIX-HFO was highest (99.1%) at pH 4 but decreased slightly at pH 5. This observation could imply the reduction of Cr(VI) ions to Cr(III) at higher pH values, and HAIX-HFO could not remove Cr(III) [58]. Another reason could be that there were more hydroxyl ions (OH^−^) in the solution at higher pH values, and they competed with Cr(VI) for the adsorption sites on HAIX-HFO [59]. Hua and colleagues [43] also found that the removal efficiency of Cr(VI) with an anionic adsorbent decreased as the pH increased from 3 to 9.

Adsorption depends on the pH of a solution, as it affects the electric charges of the adsorbent, the metal ions, and the other solutes [60,61]. As Figure 3 shows, pH affects the removal of Cr(VI) via HAIX-HFO and the removal of Cd(II) and Pb(II) via HCIX-HFO in the absence of sulfate ions. Moreover, the solution’s pH determines chromium’s oxidation state, which can either be Cr(VI) or Cr(III). In acidic solutions (pH < 5), Cr(VI) predominantly exists in HCrO_4_^−^ or Cr_2_O_7_^2−^ ion forms [62], which have a negative charge and can be adsorbed via HAIX-HFO, an anionic hybrid exchanger. On the other hand, Cr(III) has a positive charge and forms Cr^3+^ ions. Therefore, only anionic adsorbents, such as HAIX-HFO, can adsorb Cr(VI), while only cationic adsorbents, such as HCIX-HFO, can adsorb Cr(III). Hence, it is crucial to identify the type of chromium in the solution before choosing a suitable hybrid exchanger.

HCIX-HFO is a hybrid exchanger that removes positively charged metal ions like Cd(II) and Pb(II) and retains them on its surface. Cd(II) was mostly removed via HCIX-HFO at pH 3 to 4 (98.2%). Different studies have reported different effects of pH on Cd(II) adsorption using cationic adsorbents. Some studies found that Cd(II) adsorption was highest at pH 8 to 9 [63], at pH 4 to 7 [64], or at pH 4 to 5 [65,66]. These differences could be due to the type of adsorbent, Cd(II) concentration, or other molecules in the water. One possible explanation for the low adsorption of Cd(II) below pH 4 is that there were too many hydrogen ions (H^+^) in the water, and they competed with Cd(II) for the adsorption sites on HCIX-HFO. Another possible explanation for the low adsorption of Cd(II) above pH 5 is that Cd(II) changed its form and became less likely to stick to HCIX-HFO. For example, Cd(II) could form CdHCO^3+^ with bicarbonate ions [67], or it could undergo hydrolysis and form Cd(OH)^+^ or Cd(OH)_2_ [65]. Streat et al. [68] reported that HFO, a component of HCIX-HFO, could also remove Cd(II) via adsorption at pH 4 to 9 because HFO has a negative charge.

The most immobilized Pb(II) via HCIX-HFO occurred at pH 4 to 5 (97.1–97.8%). Vergili and co-workers [69] reported similar results; they found that Pb(II) adsorption was highest at pH 4 to 9. They explained that Pb(II) could exist in different forms depending on the pH, such as Pb^2+^, Pb(OH)^+^, and Pb(OH)_2_, in the pH range of 2–7. The main form of Pb(II) below pH 5 was Pb^2+^, which HCIX-HFO could remove through an ion-exchange mechanism [70]. Ahmetli et al. [71] also stated that Pb(II) was mainly in the form of Pb^2+^ in acidic water. Pehlivan and Altun [63], as well as Liu and Erhan [72], observed that Pb(II), along with other metal ions, was poorly adsorbed at pH below 4 because there were too many H^+^ ions in the water, and they displaced Pb(II) from HCIX-HFO. Dizge et al. [73] also suggested that H^+^ ions made HCIX-HFO more positive and prevented Pb(II) from approaching it. Chanthapon et al. [74] conducted their study of Pb(II) adsorption at pH 5 (±0.5). In this study, pH 4 was chosen for further experiments on Cr(VI), Cd(II), and Pb(II) adsorption to investigate other factors and conditions.

#### 3.2.2. Effects of Sulfate on Cr(VI) Adsorption

**Figure 4:**
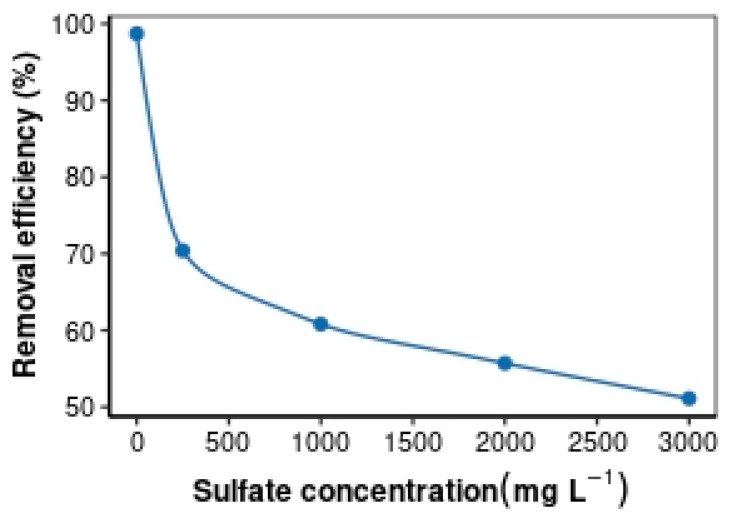
Effects of sulfate concentration on the removal of Cr(VI) via HAIX-HFO.

This study investigated how different levels of sulfate ions affect the adsorption of Cr(VI) via HAIX-HFO. It was found that as the sulfate concentration increased from 0 to 1000 mg·L^−1^, the amount of Cr(VI) adsorbed via HAIX-HFO dropped sharply to 60.8% (as seen in Figure 4). This is because sulfate ions compete with Cr(VI) for the ion-exchange sites on the resin, where replacement of chloride ions by either sulfate or Cr(VI) occurs. Increasing the sulfate concentration to 3000 mg·L^−1^ caused a significant reduction in the Cr(VI) adsorbed via HAIX-HFO to 50.8%. The SO_4_^2−^ increase created more competition for the limited adsorption sites on HAIX-HFO, resulting in a lower affinity for Cr(VI) binding. This observation entails that Cr(VI) can still bind to the iron oxide nanoparticles through ligand exchange, where hydroxyl groups become replaced by Cr(VI). Sulfate ions do not bind well to iron oxide nanoparticles, so they do not affect the ligand-exchange process [75]. HAIX-HFO possesses two kinds of adsorption sites that have different adsorption mechanisms. These are the fixed quaternary ammonium ion-exchange sites of ion-exchange resin and the ligand-exchange sites of the embedded HFO. The adsorption mechanism of quaternary ammonium groups occurs through ion exchange, where Cl^−^ counter-ions are exchanged for Cr(VI) or SO_4_^2−^. The HFO nanoparticles adsorb by way of a ligand-exchange mechanism. Blaney and co-workers [75] reported that sulfates are preferentially adsorbed on quaternary ammonium groups while forming a weak outer sphere of complexes with HFO.

#### 3.2.3. Effects of Contact Time

**Figure 5:**
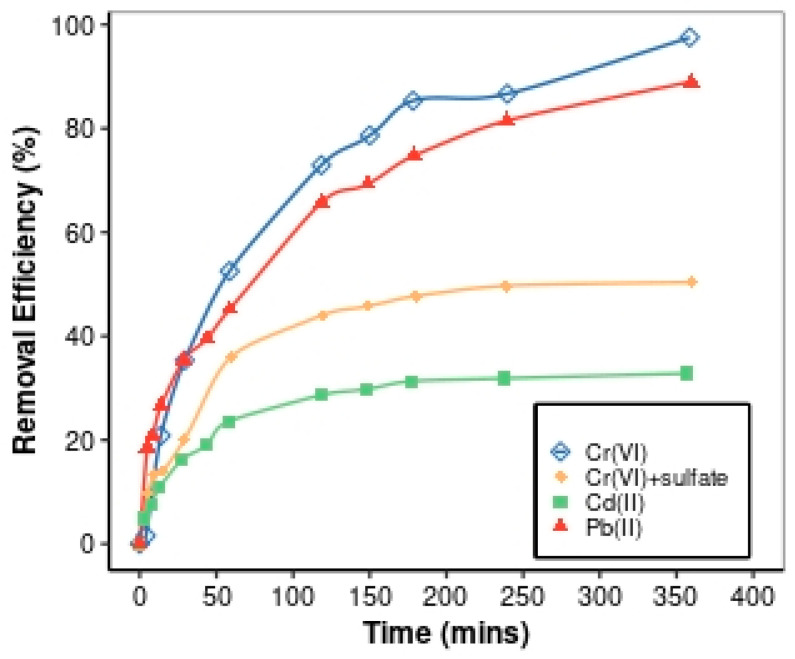
Removal efficiency for Cr(VI) with and without sulfate via HAIX-HFO as well as Cd(II) and Pb(II) in sulfate via HCIX-HFO as a function of time.

The time needed to saturate the adsorption sites influences the removal of Cr(VI), Cd(II), and Pb(II) via the respective hybrid ion-exchange resin embedded with HFO. Therefore, the contact time required to attain equilibrium was determined and thus achieve maximum adsorption of Cr(VI) ions via HAIX-HFO and Cd(II) and Pb(II) ions via HCIX-HFO, respectively. The Cr(VI) removal in the absence of sulfate was appreciably fast in the first 180 min (see Figure 5). The adsorption slowed substantially after 180 min. However, the adsorption did not reach an equilibrium within 360 min. In the presence of sulfate, a rapid adsorption of Cr(VI) occurred only within the first 60 min, and then the adsorption rate slowed down in the range of 60–180 min, taking it 240 min to reach equilibrium. The fast reaction rate enhanced via HFO nanoparticles [76] may explain the short time required to reach an equilibrium for Cr(VI) removal with co-competing sulfates. Without SO_4_^2−^ ions, both quaternary ammonium-exchange sites and HFO ligand-exchange sites adsorbed Cr(VI). This condition increased the capacity to remove Cr(VI) due to the absence of competition at either adsorption site. However, the contact time required for Cr(VI) adsorption was longer in the absence of sulfate due to adsorption at the anion-exchange sites contained within the micropores of the resin bead.

#### 3.2.4. Effects of Resin Dosage

**Figure 6:**
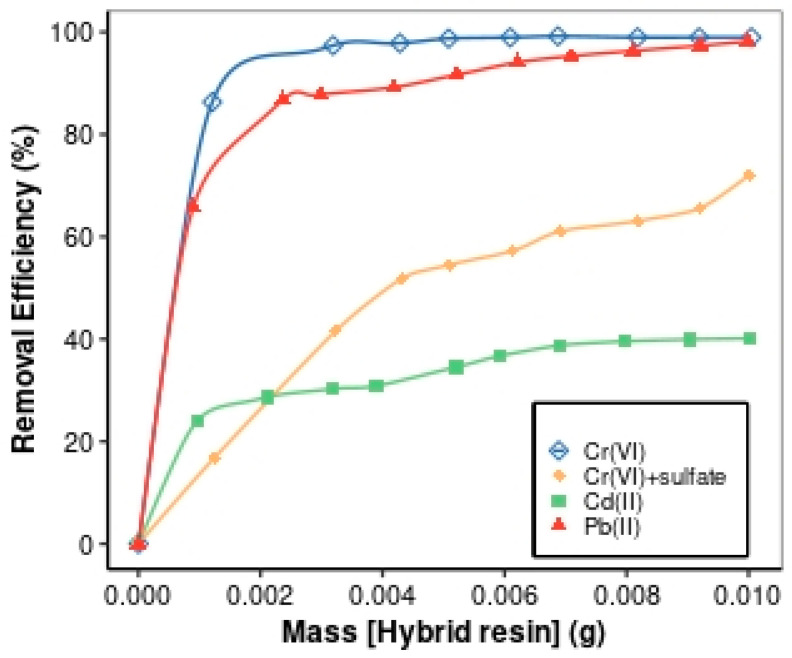
Removal efficiency for Cr(VI) with and without sulfate via HAIX-HFO as well as Cd(II) and Pb(II) via HCIX-HFO (in the presence of sulfate) as a function of resin dosage.

The efficiency of removal of contaminants depends on the adsorbent dosage. Figure 6 shows the influences of HAIX-HFO dosage on Cr(VI) adsorption with and without sulfate and the effects of HCIX-HFO dosage on Cd(II) and Pb(II) adsorption at pH 4. For Cr(VI), the rate of Cr(VI) removal increased as the dose of HAIX-HFO increased. A dosage of 0.005 g of HAIX-HFO removed almost 100% of 1 mg·L^−1^ Cr(VI) without sulfate. However, to achieve maximum Cr(VI) removal of 72.1% for 1 mg·L^−1^ Cr(VI) with 3000 mg·L^−1^ sulfate, a much higher mass of 0.01 g of HAIX-HFO was needed. This observation was due to sulfate and Cr(VI) competing for the anion-exchange sites, so more HAIX-HFO was required. More HFO adsorption sites became available as the HAIX-HFO dosage increased. For Cd(II), the adsorption amount increased slightly from 0.001 g to 0.004 g and then increased gradually. No significant change in Cd(II) removal occurred on the application of dosages exceeding 0.007 g of HCIX-HFO.

This observation was unexpected since higher dosages would provide more Cd(II) adsorption sites. For Pb(II), adsorption increased as the adsorbent dosage increased until there was no noticeable change at a mass of 0.003 g. A significant increase in adsorption occurred between 0.001 g and 0.003 g of HCIX-HFO due to the availability of more adsorption sites. Upon adding >0.003 g HCIX-HFO, approximately 90% of Pb(II) was removed from the solution. However, the residual Pb(II) ion concentration in the bulk solution was too low to maintain a strong concentration gradient that would facilitate the diffusion of Pb(II) into the pores of the resin. Chanthapon et al. [74] observed a similar pattern for adsorbent dosage in studies related to adsorption procedures using ion-exchange resins. In a different study, Kumari et al. [77] observed an elevated Cr(VI) removal with increasing chitosan dosage.

#### 3.2.5. Adsorption of Cd(II) and Pb(II) from Binary Solution

The investigation on the impact of sulfate on simultaneous Cd(II) and Pb(II) adsorption via HCIX-HFO at pH 4 showed substantial adsorption of both metals. Pb(II) exhibited a higher capacity of 2.50 mg·g^−1^ compared to Cd(II) with 1.54 mg·g^−1^ (Table 3). Interestingly, these values were similar to the singular adsorption of each metal, indicating no meaningful competitive effect. The distinct adsorption sites and mechanisms of HCIX-HFO could account for this. The ionic radius of these metals follows the order of Cd(II) < Pb(II) [78]. Cd(II), being smaller in ionic radius than Pb(II), may prefer the cation-exchange sites of the parent resin, where it faces competition from sulfate. Conversely, Pb(II) may form stable complexes with the HFO ligands through Lewis acid interactions [74]. As previously reported, HCIX-HFO demonstrated selective adsorption for Cd(II) and Pb(II) [79,80]. Kesenci et al. [81] produced a copolymer of poly(ethylene glycol dimethacrylate-acrylamide) for the removal of Pb, Hg, and Cd aqueous solutions, revealing a preferential order of metal removal as Pb > Cd > Hg.

Most Pb(II) ions tend to precipitate as PbSO_4_ in aqueous environments characterized by high sulfate concentrations. Consequently, when addressing AMD with elevated sulfate contents, the Pb(II) concentration may be significantly low. However, it is imperative to recognize that lead poses a severe health risk to humans and animals, even at low concentrations. The World Health Organization underscores that there is no safe limit for lead exposure, highlighting the importance of mitigating its presence in water sources.

#### 3.2.6. Adsorption of Metal Ions from Actual AMD

The pH and metal ion concentrations of AMD sample from the Witwatersrand Mining Basin were measured (Table 2). Using HCIX-HFO, the adsorption efficiencies for Cu(II), Ni(II), and Pb(II) were 92.5%, 92.6%, and 45.2%, respectively (Table 4). However, the adsorption of other cationic species was negligible, indicating that HCIX-HFO could not effectively remove other metal ions from the AMD sample with a low pH of 2.62. Numerous studies have demonstrated that the adsorption efficiency of heavy metal ions is notably higher under moderate pH conditions compared to lower pH levels. Razzaz et al. [82] elucidated that the maximum adsorption of Pb (II) and Cu (II) onto chitosan/TiO_2_ nanofibers occurred at pH 6.0. In contrast, the adsorption was lowest within the pH range of 2.0 to 4.0.

#### 3.2.7. Regeneration of HCIX-HFO

Regeneration experiments were conducted with NaCl and NaOH for HCIX-HFO, which had adsorbed Pb(II) and Cd(II). NaCl effectively desorbed metal ions, with removal efficiencies of 99.9% for Cd(II) and 98.8% for Pb(II). On the other hand, NaOH did not measurably desorb any metal ions. The resin manufacturer recommends the regeneration of cation-exchange resin (HCIX) that forms part of HCIX-HFO using NaCl, HCl, or H_2_SO_4_. These regeneration agents should restore the sulfonate-exchange sites to Na^+^ or H^+^ forms. NaCl and NaOH produce the Na^+^ form, while HCl and H_2_SO_4_ produce the H^+^ form. It is interesting to note that Kunaschk et al. [83] introduced an alternative perspective detailing the successful regeneration of HFO by utilizing NaOH.

#### 3.2.8. Data Modeling

The adsorption kinetics of Cr(VI) via HAIX-HFO were analyzed using three models: pseudo-first-order, pseudo-second-order, and intraparticle diffusion. The pseudo-second-order model (Table 5) provided the best fit for the experimental data in the presence and absence of sulfate. This observation suggests that chemisorption dominates the adsorption process, which involves transferring or sharing electrons between Cr(VI) and the functional groups of HAIX-HFO [32]. The efficacy between the experimental and calculated q*_e_* values from the pseudo-second-order model supports this conclusion. Kolodynska and colleagues [44] reported similar findings for Cr(VI) adsorption using commercial HFO anion-exchange resins. However, some studies have found that the pseudo-first-order model was more suitable for describing Cr(VI) adsorption kinetics in binary solutions [43,45]. The intraparticle diffusion model showed that the Cr(VI) diffusion rate was higher without sulfate than with sulfate (Table 5). This revelation indicates that the presence of sulfate on the surface of HAIX-HFO hindered the diffusion of Cr(VI) into the pores of the adsorbent [84]. The boundary layer thickness (BLT) values also confirmed this effect, as they were lower without sulfate than with sulfate.

The adsorption kinetics of Cd(II) and Pb(II) via HCIX-HFO were also described well with the pseudo-second-order model, as shown by the similarity between the experimental and calculated q*_e_* values. The model has also been reported to fit the adsorption data of Cd(II) with sulfonate cation-exchange resins [64] and with cationic inodiacetate-exchange resins [66]. The intraparticle diffusion model indicated that Cd(II)’s and Pb(II)’s surface adsorption on HCIX-HFO was negligible, as evidenced by the very low or zero BLT values.

The equilibrium isotherms provide insight into the adsorption mechanism. Three parameter models (Langmuir, Freundlich, and Temkin) were fitted to the isotherm data (Table 6). The modeled adsorption capacities (q*_max_*) were compared to the experimentally determined adsorption capacities (q*_e·exp_*) and the calculated capacities (q*_e·ca_*_l_) from each model. The Langmuir model, which assumes monolayer adsorption with uniform binding sites and energies [85], provided the best fit for Cr(VI) adsorption without sulfate. This case’s Langmuir separation factor (RL) was 0.0467, indicating favorable adsorption [86]. The Temkin model, which considers the interactions between adsorbate and adsorbent as well as the variation of binding energies, was more suitable for Cr(VI) adsorption with sulfate.

The adsorption equilibrium of Cd(II) using HCIX-HFO needed to be better represented by any of the three models. The Freundlich and Langmuir models were the closest but had some limitations. The Freundlich model had an n value of 0.35, which implied unfavorable adsorption of Cd(II). This outcome suggested that HCIX-HFO had a low Cd(II) adsorption capacity. The Langmuir model had negative values for K*_L_* and Q*_max_*, which were not physically meaningful [87].

The Freundlich model best described the adsorption equilibrium of Pb(II) using HCIX-HFO at pH 4 with a sulfate medium. The Freundlich model parameters, K*_F_* and n, were 17.6 L·g^−1^ and 1.73, respectively. The n value 1.73 indicated favorable Pb(II) adsorption, while the K*_F_* value showed strong adsorption. Chanthapon et al. [74] reported a similar Pb(II) adsorption result on a cationic exchanger with zerovalent iron nanoparticles. The Freundlich model fitted the experimental data closely at low Pb(II) concentrations, while the Langmuir model was more suitable at high concentrations. This finding could be due to the different types of adsorption sites in HCIX-HFO, such as the ligand-exchange sites of the ferric oxide nanoparticles and the sulfonate cation-exchange sites of the resin.

## 4. Conclusions

This study entailed developing hybrid ion-exchange resins by integrating HFO nanoparticles into anionic and cationic resin matrices. The anionic hybrid resin, HAIX-HFO, showed remarkable performance in removing Cr(VI) from acidic water, achieving almost a 99.1% adsorption efficiency at pH 4 and a slightly lower one at pH 5. Despite the high sulfate concentration, which reduced Cr(VI) adsorption, HAIX-HFO exhibited selectivity for Cr(VI). The adsorption kinetics of Cr(VI) were explained via the pseudo-second-order and intraparticle diffusion models, the applicability of which varied depending on sulfate presence or absence. The R^2^ values for the pseudo-second-order values were 0.9929 and 0.9856 for Cr(VI) with and without sulfate, respectively. The intraparticle diffusion model revealed that sulfate on the HAIX-HFO surface lowered the Cr(VI) diffusion rate. Furthermore, the Langmuir and Temkin models were the best fit for describing the adsorption equilibrium of Cr(VI) without and with sulfate, respectively. Similarly, the cationic hybrid resin, HCIX-HFO, demonstrated optimal adsorption efficiencies of 98.2% and 97.1% at pH 4 for Cd(II) and Pb(II), respectively. The pseudo-second-order model described their adsorption kinetics in sulfate. While Cd(II) adsorption equilibrium followed the Freundlich and Langmuir models, the Freundlich model indicated that HCIX-HFO may not be a suitable Cd(II) adsorbent. In the binary solution, HCIX-HFO preferred Pb(II) over Cd(II). Furthermore, HCIX-HFO displayed its effectiveness in removing Cu(II), Ni(II), and Pb(II) from an actual acid mine drainage sample. Moreover, the use of NaCl demonstrated exceptional capability in regenerating heavy metal-laden HCIX-HFO. These findings underscore the potential of hybrid ion-exchange resins incorporating HFO nanoparticles in addressing the challenges associated with acid mine drainage treatment.

## Figures and Tables

**Table 1:** Properties of Amberlite IRA400 and IMAC HP1110 unmodified resins.

	IRA400	HP1110
Notation	HAIX	HCIX
Matrix	Polystyrene/divinylbenzene copolymer	Styrene/divinylbenzene (gel)
Functional groups	Quaternary ammonium	Sulfonates
Total exchange capacity	≥1.4 eq/L	≥2.2 eq/L
Moisture holding capacity	40–47%	36–44%
Ionic form	Cl^−^	Na^+^
Physical form	Pale yellow translucent beads	Uniform size, amber beads

**Table 2:** Mean concentration of heavy metals in AMD.

Metal	Concentration (mg·L^−1^)
Cd	0.021 (±1.21 × 10^−6^)
Co	3.147 (±0.027)
Cr	0.188 (±4.22 × 10^−6^)
Cu	1.421 (±3.59 × 10^−5^)
Fe	0.910 (±0.0002)
Mg	31.20 (±0.041)
Ni	16.10 (±0.052)
Pb	0.031 (±2.85 × 10^−6^)
Ti	20.53 (±1.75)
Zn	14.70 (±0.055)

**Table 3:** Mean Cd(II) and Pb(II) removal using HCIX-HFO.

Metal Ion	Removal Efficiency (%)	Adsorption Capacity, q*_e_* (mg·g^−1^)
Pb(II)	99.67 (±0.032)	2.50 (±0.011)
Cd(II)	30.54 (±0.029)	1.54 (±0.050)

**Table 4:** Adsorption of metal ions from the actual AMD.

	AMD before	AMD after	Removal	Adsorption
	Treatment (mg·L^−1^)	Adsorption (mg·L^−1^)	Efficiency (%)	Capacity (mg·L^−1^)
Cu	1.421 (±3.59 × 10^−5^)	0.107 (±7.58 × 10^−6^)	92.47 (±0.194)	0.522 (±0.0135)
Ni	16.10 (±0.052)	1.192 (±3.33 × 10^−5^)	92.60 (±0.036)	5.86 (±0.0283)
Pb	0.031 (±2.85 × 10^−6^)	0.017 (±1.23 × 10^−11^)	45.16 (±0.011)	0.069 (±1.72 × 10^−5^)

**Table 5:** Kinetics model parameters for Cr(VI) adsorption on HAIX-HFO as well as Cd(II) and Pb(II) adsorption on HCIX-HFO in pH 4 aqueous solution.

				Pseudo-First-Order Model			Pseudo-Second-Order Model			Intraparticle		
Hybrid	Metal Ion	SO_4_^2−^	q*_e·exp_*	K_1_	q*_e1·cal_*	R^2^	K_2_	q*_e2·cal_*	R^2^	K*_ip_*	BLT	R^2^
Sorbent	(mg·L^−1^)	(mg·L^−1^)	(mg·g^−1^)	(min^−1^)	(mg·g^−1^)	(-)	(g·min^−1^mg^−1^)	(mg·g^−1^)	(-)	(mg·g^−1^min^0.5^)	(-)	(-)
HAIX-HFO	Cr(VI)	0	4.91	0.006	2.970	0.5784	0.002	5.79	0.9856	0.4323	0.0806	0.9735
HAIX-HFO	Cr(VI)	3000	2.22	0.006	1.020	0.1898	0.013	2.47	0.9929	0.1524	0.1045	0.9794
HCIX-HFO	Pb(II)	3000	1.45	0.006	0.642	0.2249	0.016	1.65	0.9964	0.1282	0.0611	0.9858
HCIX-HFO	Cd(II)	3000	2.27	0.004	1.200	0.3749	0.008	2.54	0.9773	0.1123	0.2606	0.9874

**Table 6:** Freundlich, Langmuir, and Temkin isotherm constants for the adsorption of Cr(VI) on HAIX-HFO as well as Cd(II) and Pb(II) on HCIX-HFO.

			Freundlich			Langmuir			Temkin		
Hybrid	Metal Ion	SO_4_^2−^	K*_F_*	n	R^2^	Q*_max_*	K*_L_*	R^2^	A	B	R^2^
Sorbent	(mg·L^−1^)	(mg·L^−1^)	(L·g^−1^)	(-)	(-)	(mg·g^−1^)	(L·mg^−1^)	(-)	(L·g^−1^)	(J·mol^−1^)	(-)
HAIX-HFO	Cr(VI)	0	61.20	1.70	0.9643	25.10	18.14	0.9764	223	494	0.9635
HAIX-HFO	Cr(VI)	3000	4.48	1.30	0.8438	8.88	0.83	0.4218	8.90	1357	0.8505
HCIX-HFO	Cd(II)	3000	94.00	0.35	0.8472	−1.79	−2.00	0.8385	4.52	208	0.6472
HCIX-HFO	Pb(II)	3000	17.60	1.73	0.8801	13.76	6.86	0.5158	152	1305	0.7261

## Data Availability

The raw data supporting the conclusions of this article will be made available by the authors upon request.

## References

[1] Kefeni K.K., Mamba B.B. (2021). Charcoal ash leachate and its sparingly soluble residue for acid mine drainage treatment: Waste for pollution remediation and dual resource recovery. J. Clean. Prod..

[2] Tabelin C.B., Uyama A., Tomiyama S., Villacorte-Tabelin M., Phengsaart T., Silwamba M., Jeon S., Park I., Arima T., Igarashi T. (2022). Geochemical audit of a historical tailings storage facility in Japan: Acid mine drainage formation, zinc migration and mitigation strategies. J. Hazard Mater..

[3] Aguilar-Garrido A., Paniagua-Lopez M., Sierra-Aragon M., Garzon F.J.M., Martın-Peinado F.J. (2023). Remediation potential of mining, agro-industrial, and urban wastes against acid mine drainage. Sci. Rep..

[4] Thomas G., Sheridan C., Holm P.E. (2022). A critical review of phytoremediation for acid mine drainage-impacted environments. Sci. Total Environ..

[5] Hussain S., Bharali P., Koushik B., Hoque R.R. (2022). Bioaccumulation of metals in lichens and mosses understanding atmospheric deposition, metal-induced modifications and their suitability as biomonitors and bioremediators. Bioremediation of Toxic Metal(loid)s.

[6] Balali-Mood M., Naseri K., Tahergorabi Z., Khazdair M.R., Sadeghi M. (2021). Toxic mechanisms of five heavy metals: Mercury, lead, chromium, cadmium, and arsenic. Front. Pharmacol..

[7] Masindi V., Foteinis S., Renforth P., Ndiritu J., Maree J., Tekere M., Chatzisymeon E. (2022). Challenges and avenues for acid mine drainage treatment, beneficiation, and valorisation in circular economy: A review. Ecol. Eng..

[8] Zeng J., Qiu J., Zhang J., Qi Y., Liu R., Jian C., Liu N., Su Y. (2023). Plant ash prevents acid mine drainage from sulfur-bearing tailings through multiple actions —A low-cost alkaline material. Appl. Geochem..

[9] Mwewa B., Tadie M., Ndlovu S., Simate G.S., Matinde E. (2022). Recovery of rare earth elements from acid mine drainage: A review of the extraction methods. J. Environ. Chem. Eng..

[10] Alkhadra M.A., Su X., Suss M.E., Tian H., Guyes E.N., Shocron A.N., Conforti K.M., Souza J.P., Kim N., Tedesco M. (2022). Electrochemical methods for water purification, ion separations, and energy conversion. Chem. Rev..

[11] Rambabu K., Banat F., Pham Q.M., Ho S.H., Ren N.Q., Show P.L. (2020). Biological remediation of acid mine drainage: Review of past trends and current outlook. Environ. Sci. Ecol..

[12] Yuan J., Ding Z., Bi Y., Li J., Wen S., Bai S. (2022). Resource utilization of acid mine drainage (AMD): A review. Water.

[13] Chostak C.L., Lopez-Delgado A., Padilla I., Lapolli R.F., Lobo-Recio M.A. (2023). Use of a waste-derived linde type-a immobilized in agarose for the remediation of water impacted by coal acid mine drainage at pilot scale. Materials.

[14] Li R., Wang B., Wu P., Zhang J., Zhang X., Chen M., Cao X., Feng Q. (2023). Revealing the role of calcium alginate-biochar composite for simultaneous removing SO_4_^2−^ and Fe^3+^ in AMD: Adsorption mechanisms and application effects. Environ. Pollut..

[15] Cyganowski P., Dzimitrowicz A. (2020). A Mini-Review on Anion Exchange and Chelating Polymers for Applications in Hydrometallurgy, Environmental Protection, and Biomedicine. Polymers.

[16] Liao J., He X., Zhang Y., Zhang L., He Z. (2023). The construction of magnetic hydroxyapatite-functionalized pig manure-derived biochar for the efficient uranium separation. Chem. Eng. J..

[17] Pyrzynska K. (2019). Removal of cadmium from wastewaters with low-cost adsorbents. J. Environ. Chem. Eng..

[18] Zaharia M.M., Bucatariu F., Vasiliu A.L., Mihai M. (2022). Stable and reusable acrylic ion-exchangers from HMIs highly polluted tailing pond to safe and clean water. Chemosphere.

[19] Ahmaruzzaman M. (2011). Industrial wastes as low-cost potential adsorbents for the treatment of wastewater laden with heavy metals. Adv. Colloid Interface Sci..

[20] Fei Y., Hu Y.H. (2022). Design, synthesis, and performance of adsorbents for heavy metal removal from wastewater: A review. J. Mater. Chem. A.

[21] Phiri Z., Moja N.T., Nkambule T.T.I., de Kock L.A. (2024). Utilization of biochar for remediation of heavy metals in aqueous environments: A review and bibliometric analysis. Heliyon.

[22] Liu B., Vikrant K., Kim K.-H., Kumar V., Kailasa S.K. (2020). Critical role of water stability in metal–organic frameworks and advanced modification strategies for the extension of their applicability. Environ. Sci. Nano.

[23] Whitworth T.M. (1998). Clay minerals: Ion exchange. Geochemistry. Encyclopedia of Earth Science.

[24] Derbe T., Temesgen S., Bitew M. (2021). A short review on synthesis, characterization, and applications of zeolites. Adv. Mater. Sci. Eng..

[25] Alhalili Z. (2023). Metal oxides nanoparticles: General structural description, chemical, physical, and biological synthesis methods, role in pesticides and heavy metal removal through wastewater treatment. Molecules.

[26] Hassanzadeh-Afruzi F., Esmailzadeh F., Asgharnasl S., Ganjali F., TaheriLedari R., Maleki A. (2022). Efficient removal of Pb(II)/Cu(II) from aqueous samples by a guanidine-functionalized SBA-15/Fe_3_O_4_. Sep. Purif. Technol..

[27] Ma Q., Teng W., Sun Y., Chen Y., Xue Y., Chen X., Zhang C., Zhang H., Fan J., Qiu Y. (2022). Multi-component removal of Pb(II), Cd(II), and As(V) over core-shell structured nanoscale zero-valent iron@mesoporous hydrated silica. Sci. Total Environ..

[28] Rather M.A., Bhuyan S., Chowdhury R., Sarma R., Roy S., Neog P.R. (2023). Nanoremediation strategies to address environmental problems. Sci. Total Environ..

[29] Borji H., Ayoub G.M., Bilbeisi R., Nassar N., Malaeb L. (2020). How effective are nanomaterials for the removal of heavy metals from water and wastewater?. Water Air Soil Pollut..

[30] Wang L., Xu H., Qiu Y., Liu X., Huang W., Yan N., Qu Z. (2020). Utilization of Ag nanoparticles anchored in covalent organic frameworks for mercury removal from acidic waste water. J. Hazard Mater..

[31] Gregor H.P. (1951). Gibbs-Donnan equilibria in ion exchange resin systems. J. Am. Chem. Soc..

[32] SenGupta A.K. (2017). Ion Exchange in Environmental Processes: Fundamentals, Applications and Sustainable Technology.

[33] Muhammad A., Soares A., Jefferson B. (2019). The impact of background wastewater constituents on the selectivity and capacity of a hybrid ion exchange resin for phosphorus removal from wastewater. Chemosphere.

[34] Perlova O., Dzyazko Y., Halutska I., Perlova N., Palchik A. (2018). Anion exchange resin modified with nanoparticles of hydrated zirconium dioxide for sorption of soluble U(VI) compounds. Nanooptics, Nanophotonics, Nanostructures, and Their Applications.

[35] Manjare S.B., Chaudhari R.A., Thopate S.R., Risbud K.P., Badade S.M. (2020). Resin loaded palladium nanoparticle catalyst, characterization and application in –C–C– coupling reaction. SN Appl. Sci..

[36] Martin B.D., Parsons S.A., Jefferson B. (2009). Removal and recovery of phosphate from municipal wastewaters using a polymeric anion exchanger bound with hydrated ferric oxide nanoparticles. Water Sci. Technol..

[37] Beaundry J.W., Sengupta S. (2021). Phosphorus recovery from wastewater using pyridine-based ion-exchange resins: Role of impregnated iron oxide nanoparticles and preloaded Lewis acid (Cu^2+^). Water Environ. Res..

[38] You X., Valderrama C., Soldatov V., Cortina J.L. (2018). Phosphate recovery from treated municipal wastewater using hybrid anion exchangers containing hydrated ferric oxide nanoparticles. J. Chem. Technol. Biotechnol..

[39] Gifford M., Chester M., Hristovski K., Westerhoff P. (2016). Reducing environmental impacts of metal (hydr)oxide nanoparticle embedded anion exchange resins using anticipatory life cycle assessment. Environ. Sci. Nano.

[40] Nguyen T.T., Vu Anh Khoa Tran V.A.K., Tran L.B., Phan P.T., Nguyen M.T., Bach L.G., Padungthon S., Ta C.K., Nguyen N.H. (2021). Synthesis of cation exchange resin-supported iron and magnesium oxides/hydroxides composite for nitrate removal in water. Chin. J. Chem. Eng..

[41] Maltseva T.V., Kolomiets E.O., Dzyazko Y.S., Scherbakov S. (2019). Composite anion-exchangers modified with nanoparticles of hydrated oxides of multivalent metals. Appl. Nanosci..

[42] Acelas N.Y., Martin B.D., Lopez D., Jefferson B. (2015). Selective removal of phosphate from wastewater using hydrated metal oxides dispersed within anionic exchange media. Chemosphere.

[43] Hua M., Yang B., Shan C., Zhang W., He S., Lv L., Pan B. (2017). Simultaneous removal of As(V) and Cr(VI) from water by macroporous anion exchanger supported nanoscale hydrous ferric oxide composite. Chemosphere.

[44] Kolodynska D., Kowalczyk M., Hubicki Z., Shvets V., Golub V. (2015). Effect of accompanying ions and ethylenediaminedisuccinic acid on heavy metals sorption using hybrid materials Lewatit FO 36 and Purolite Arsen X^np^. Chem. Eng. J..

[45] Kowalczyk M., Hubicki Z., Kolodynska D. (2013). Modern hybrid sorbents – New ways of heavy metal removal from waters. Chem. Eng. Process.

[46] McCarthy T.S. (2011). The impact of acid mine drainage in South Africa. S. Afr. J. Sci..

[47] Karapınar N. (2016). Removal of heavy metal ions by ferrihydrite: An opportunity to the treatment of acid mine drainage. Water Air Soil Pollut..

[48] Zhu T., Zhang Y., Chen Y., Liu J.-L., Song X.-L. (2022). Synthesis of novel hydrated ferric oxide biochar nanohybrids for efficient arsenic removal from wastewater. Rare Met..

[49] Pan B., Wu J., Pan B., Lv L., Zhang W., Xiao L., Wang X., Tao X., Zheng S. (2009). Development of polymer-based nanosized hydrated ferric oxides (HFOs) for enhanced phosphate removal from waste effluents. Water Res..

[50] Kefeni K.K., Msagati T.A., Mamba B.B. (2017). Acid mine drainage: Prevention, treatment options, and resource recovery: A review. J. Clean. Prod..

[51] Luptakova A., Macingova E., Kotulicova I., Rudzanova D. (2016). Sulphates removal from acid mine drainage. IOP Conf. Ser. Earth Environ. Sci..

[52] De Kock L.A. (2015). Hybrid Ion Exchanger Supported Metal Hydroxides for the Removal of Phosphate from Wastewater. Ph.D. Thesis.

[53] Cornell R.M., Schwertmann U. (2003). The Iron Oxides: Structure, Properties, Reactions, Occurrences and Uses.

[54] Hanesch M. (2009). Raman spectroscopy of iron oxides and (oxy)hydroxides at low laser power and possible applications in environmental magnetic studies. Geophys. J. Int..

[55] Das S., Hendry M.J. (2011). Application of Raman spectroscopy to identify iron minerals commonly found in mine wastes. Chem. Geol..

[56] Hara E. (2022). Detecting organics with deep UV Raman and fluorescence spectroscopy. Nat. Rev. Earth Environ..

[57] Wei D., Chen S., Liu Q. (2015). Review of fluorescence suppression techniques in Raman spectroscopy. Appl. Spectrosc. Rev..

[58] Xiao K., Xu F., Jiang L., Duan N., Zheng S. (2016). Resin oxidization phenomenon and its influence factor during chromium (VI) removal from wastewater using gel-type anion exchangers. Chem. Eng. J..

[59] Polowczyk I., Urbano B.F., Rivas B.L., Bryjak M., Kabay N. (2016). Equilibrium and kinetic study of chromium sorption on resins with quaternary ammonium and N-methyl-D-glucamine groups. Chem. Eng. J..

[60] Cruz-Lopes L.P., Macena M., Esteves B., Guine R.P.F. (2021). Ideal pH for the adsorption of metal ions Cr^6+,^ Ni^2+,^ Pb^2+^ in aqueous solution with different adsorbent materials. Open Agric..

[61] Karimadom B.R., Meyerstein D., Kornweitz H. (2021). Calculating the adsorption energy of a charged adsorbent in a periodic metallic system—the case of BH_4_^−^ hydrolysis on the Ag(111) surface. Phys. Chem. Chem. Phys..

[62] Fenti A., Chianese S., Iovino P., Musmarra D., Salvestrini S. (2020). Cr(VI) sorption from aqueous solution: A review. Appl. Sci..

[63] Pehlivan E., Altun T. (2006). The study of various parameters affecting the ion exchange of Cu^2+^, Zn^2+^, Ni^2+^, Cd^2+^, and Pb^2+^ from aqueous solution on Dowex 50W synthetic resin. J. Hazard. Mater..

[64] Bai Y., Bartkiewicz B. (2009). Removal of cadmium from wastewater using ion exchange resin Amberjet 1200 h columns. Pol. J. Environ. Stud..

[65] Wang F., Jun W.L., Sheng L.J., Yun S.X., Qing H.W. (2009). Adsorption behavior and mechanism of cadmium on strong-acid cation exchange resin. Trans. Nonferrous Met. Soc. China.

[66] Wong C.W., Barford J.P., Chen G., McKay G. (2014). Kinetics and equilibrium studies for the removal of cadmium ions by ion exchange resin. J. Environ. Chem. Eng..

[67] Mislin H., Ravera O. (1986). Cadmium in the Environment.

[68] Streat M., Hellgardt K., Newton N. (2008). Hydrous ferric oxide as an adsorbent in water treatment. Process Saf. Environ. Prot..

[69] Vergili I., Soltobaeva G., Kaya Y., Gönder Z.B., Cavus S., Gürdag G. (2013). Study of the removal of Pb(II) using a weak acidic cation resin: Kinetics, thermodynamics, equilibrium, and breakthrough curves. Ind. Eng. Chem. Res..

[70] Noeline B., Manohar D., Anirudhan T. (2005). Kinetic and equilibrium modelling of lead (II) sorption from water and wastewater by polymerized banana stem in a batch reactor. Sep. Purif. Technol..

[71] Ahmetli G., Yel E., Deveci H., Bravo Y., Bravo Z. (2011). Investigation of Pb(II) adsorption onto natural and synthetic polymers. J. Appl. Polym. Sci..

[72] Liu Z.S., Erhan S.Z. (2002). Conversion of soybean oil into ion exchange resins: Removal of copper (II), nickel (II), and cobalt (II) ions from dilute aqueous solution using carboxylate-containing resin. J. Appl. Polym..

[73] Dizge N., Keskinler B., Barlas H. (2009). Sorption of Ni(II) ions from aqueous solution by Lewatit cation-exchange resin. J. Hazard. Mater..

[74] Chanthapon N., Sarkar S., Kidkhunthod P., Padungthon S. (2018). Lead removal by a reusable gel cation exchange resin containing nano-scale zero valent iron. J. Chem. Eng..

[75] Blaney L., Cinar S., SenGupta A. (2007). Hybrid anion exchanger for trace phosphate removal from water and wastewater. Water Res..

[76] Gautam R.K., Sharma S.K., Mahiya S., Chattopadhyaya M.C. (2014). Chapter 1. contamination of heavy metals in aquatic media: Transport, toxicity and technologies for remediation. Heavy Metals in Water.

[77] Kumari S., Rath P., Kumar A.S.H., Tiwari T.N. (2016). Removal of hexavalent chromium using chitosan prepared from shrimp shells. Afr. J. Biotechnol..

[78] Macanas J., Ruiz P., Alonso A., Muñoz M., Muraviev D. (2011). Ion exchange-assisted synthesis of polymer stabilized metal nanoparticles. Ion Exchange and Solvent Extraction Series.

[79] Guo S., Dan Z., Duan N., Chen G., Gao W., Zhao W. (2018). Zn(II), Pb(II), and Cd(II) adsorption from aqueous solution by magnetic silica gel: Preparation, characterization, and adsorption. Environ. Sci. Pollut. Res..

[80] Jazi M.B., Arshadi M., Amiri M., Gil A. (2014). Kinetic and thermodynamic investigations of Pb(II) and Cd(II) adsorption on nanoscale organofunctionalized SiO_2_-Al_2_O_3_. J. Colloid Interface Sci..

[81] Kesenci K., Say R., Denizli A. (2002). Removal of heavy metal ions from water by using poly (ethyleneglycol dimethacrylate-co-acrylamide) beads. Eur. Polym. J..

[82] Razzaz A., Ghorban S., Hosayni L., Irani M., Aliabadi M. (2016). Chitosan nanofibers functionalized by TiO2 nanoparticles for the removal of heavy metal ions. J. Taiwan Inst. Chem. Eng..

[83] Kunaschk M., Schmalz V., Dietrich N., Dittmar T., Worch E. (2015). Novel regeneration method for phosphate loaded granular ferric (hydr)oxide – a contribution to phosphorus recycling. Water Res..

[84] Koushkbaghi S., Zakialamdari A., Pishnamazi M., Ramandi H.F., Aliabadi M., Irani M. (2018). Aminated-Fe_3_O_4_ nanoparticles filled Chitosan/PVA/PES dual layers nanofibrous membrane for the removal of Cr(VI) and Pb(II) ions from aqueous solutions in adsorption and membrane processes. Chem. Eng. J..

[85] Al-Ghouti M.A., Da’ana D.A. (2020). Guidelines for the use and interpretation of adsorption isotherm models: A review. J. Hazard. Mater..

[86] Worch E. (2021). Adsorption Technology in Water Treatment.

[87] Alshabanat M., Alsenani G., Almufarij R. (2013). Removal of crystal violet dye from aqueous solutions onto date palm fiber by adsorption technique. J. Chem..

